# Association of common gene-smoking interactions with elevated plasma apolipoprotein B concentration

**DOI:** 10.1186/s12944-020-01287-7

**Published:** 2020-05-19

**Authors:** Nathalie Roy, Daniel Gaudet, Gérald Tremblay, Diane Brisson

**Affiliations:** 1 Department of Medicine, Université de Montréal, ECOGENE-21 Clinical and Translational Research Center, 930 Jacques-Cartier, Chicoutimi, Quebec G7H 7K9 Canada; 2 Lipid Clinic, Chicoutimi Hospital, Saguenay, Quebec Canada

**Keywords:** Apolipoprotein B, Smoking, Gene variants

## Abstract

**Background:**

Increased apolipoprotein (apo) B level (hyperapoB) is a strong predictor of cardiovascular disease (CVD), even in patients who achieve recommended LDL-Cholesterol (LDL-C) goals. ApoB level, an important correlate of metabolic syndrome (MetS), is influenced by several gene-environment interactions. Some of them are rare and can explain a large proportion of apoB variance, whereas others more common have variable effects. The aim of this study was to evaluate the association of interaction between smoking and common hyperapoB gene variants (PPARα-L162V, lipoprotein lipase loss-of function mutation, apo e4 allele or apo E2/2 genotype) with plasma apoB concentrations, according to the expression of MetS.

**Methods:**

This study was performed among 1798 subjects. Smoking was defined as non/mild smokers vs. moderate-to-heavy smokers. ApoB levels were determined using nephelometry. Logistic regression models were used to document interactions between smoking habits and the presence of hyperapoB gene variants on the relative odds to exhibit increased plasma apoB concentrations.

**Results:**

Around 29% of individuals with a low-risk lipid profile without MetS component had hyperapoB. Smoking and the presence of hyperapoB gene variants tended to be associated with higher plasma apoB levels even in presence of low-LDL-C. There was a significant interaction (*P* = 0.04) between the presence of ≥1 gene variants and smoking on the risk to exhibit hyperapoB among subjects with low risk profile in primary prevention.

**Conclusions:**

Combination of life habits assessment and some common genes variants may detect a significant proportion of patients with increased apoB levels, and therefore a higher risk of CVD, who could have been initially perceived as low-risk.

## Background

Low-density lipoprotein-cholesterol (LDL-C) and non-high-density lipoprotein-cholesterol (non-HDL-C) are well-known risk factors of cardiovascular disease (CVD) and important targets for intervention. Although the interest given to increased plasma apolipoprotein (apo) B concentrations is more recent, it has long been proven to also be an efficient marker of CVD risk in various studies and is now starting to be used as such [1–3]. In fact, the predictive power of hyperapobetalipoproteinemia (hyperapoB) would remain significant even in individuals with low-to-normal LDL-C [4].

Plasma apoB concentration is affected by a huge number of genetic factors, but most of those having a large size effect, such as familial hypercholesterolemia (FH)-causing gene variants, are not very frequent [5, 6]. However, the effect of most genetic variants is much more modest. ApoE gene polymorphisms, the peroxisome proliferator-activated receptor (PPAR)α-L162V mutation, as well as loss-of-function (LoF) lipoprotein lipase (LPL) gene mutations, are examples of common variants modulating apoB-containing lipoprotein metabolism. PPARα-L162V mutation and several LoF LPL gene variants have been associated with higher apoB levels [7, 8]. The differential contribution of common apoE polymorphisms (APOE2, 3 and 4) to apoB-containing lipoprotein metabolism is also well documented. APOE4 is associated with higher apoB, LDL-C levels and cholesterol absorption/synthesis ratio than APOE3. APOE2, in the homozygous state, is associated with a reduced very-low-density lipoprotein (VLDL)-remnants clearance, an increased number of intermediate-density lipoprotein (IDL) particles (β-VLDL) and a higher concentration of IDL-apoB, increasing the risk of dysbetalipoproteinemia and CVD [9].

ApoB is closely related to the expression of the metabolic syndrome (MetS), a worldwide increasingly prevalent phenotype highly influenced by life habits [10, 11]. While the literature abounds with studies on associations between apoB levels and life habits such as diet, alcohol and physical activity, it is less the case with smoking. According to available results, effects of smoking on lipid metabolism appear more obviously related to the HDL and triglycerides (TG) metabolism [12–15]. Fewer results target its association with apoB and these are not all in agreement [16–18].

An increasing number of gene-smoking interaction studies and transcriptome analyses have been published so that there is now abundant evidence about the interaction between smoking and gene factors in several metabolic pathways [19–21]. However, there are very few results and numerous disagreements about gene x smoking interactions influence on the lipid-lipoprotein metabolism, especially regarding apoB [22, 23]. Thus, while the relative CVD risk associated with rare monogenic conditions such as FH is recognized, the risk of different combinations of common genetic variants and environmental factors is still quite less known.

The aim of this study was to evaluate the association of interactions between smoking and common gene variants affecting apoB catabolic pathways (PPARα-L162V, heterozygous loss-of function LPL mutation, apo e4 allele or apo E2/2 genotype) with plasma apoB concentrations, according to the expression of MetS.

## Methods

### Subjects and clinical data

This study comprised a sample of 1798 French Canadians subjects from the Saguenay–Lac-Saint-Jean region of Quebec (Canada). All subjects were screened at the Chicoutimi Hospital Lipid Clinic or ECOGENE-21 Clinical Research Center and agreed to participate in studies on genetic determinants of type 2 diabetes or coronary artery disease (CAD) combining genome wide scans and candidate gene strategies. Subjects were selected to be included in the present study based on the availability of data on targeted apoB-associated mutations/polymorphisms, plasma apoB concentrations and smoking habits. Subjects with familial chylomicronemia (complete LPL deficiency) and those taking drugs known to affect blood lipid levels were excluded. Subjects were classified according to the MetS diagnosis, as confirmed by the presence of ≥3/5 of the following components: waist circumference > 102 cm in men or > 88 cm in women; fasting plasma TG concentrations ≥1.7 mmol/L; HDL-C < 1.0 mmol/L in men or < 1.3 mmol/L in women; blood pressure ≥ 130 mmHg or ≥ 85 mmHg for systolic or diastolic blood pressure, respectively, or hypertensive treatment; fasting glucose > 5.6 mmol/L or drug treatment for elevated glucose [10]. The presence of CAD was documented using patient’s medical charts on the basis of clinical and electrocardiography (ECG) criteria of myocardial infarction or evidence of coronary stenosis of at least 50% in > 1 main coronary arteries following coronary angiography for the investigation of ischemic heart disease. Waist girth was determined according to the procedures of the Airlie conference [24]. Smoking habits were classified according to the number of cigarettes smoked daily (0 to 10 (non/mild smokers) vs. more than 10 (moderate-to-heavy smokers)). Subjects gave their informed consent to participate in this study and were assigned a code that systematically de-identifies all clinical data [25]. This study has received the approval of the Chicoutimi Hospital Ethics Committee, and was conducted in accordance with the Declaration of Helsinki.

### Biochemical analysis

Blood samples were obtained after a 12-h overnight fast from the antecubital vein into vacutainer tubes containing EDTA. Cholesterol, TG and glucose levels were measured by enzymatic assays on a CX7Analyser (Beckman, Fullerton, CA, USA) [26]. Total cholesterol was determined in plasma and HDL after precipitation of VLDL and LDL (d > 1.006 g/ml) in the infranatant with heparin and manganese chloride (MnCl2). In this case, plasma LDL cholesterol levels were estimated using the Friedewald formula [27] unless TG level was higher than 4.5 mmol/L, in which case a direct measurement was used. ApoB levels were determined using nephelometry.

### Genotyping

LoF LPL gene variants included those with a combined prevalence of at least 5% in the Eastern Quebec French Canadian Founder population (P207L, G188E) or reported as prevalent worldwide (D9N and N291S). The PPARα-L162V variant, the presence of P207L, G188E, D9N and N291S variants in the LPL gene and the apo E genotype were identified by a mismatch polymerase chain reaction-restriction fragment length polymorphism (PCR-RFLP) based method, as previously described [28–33].

### Statistical analysis

Categorical variables were compared using the Pearson χ^2^ statistic, whereas group differences for continuous variables were compared with the Student’s unpaired two-tailed t–test. Log10-transformed data and medians (interquartile ranges) were used for variables with a non-normal distribution. Multivariate logistic regression models were built to document the relation between increase in the number of MetS components and the proportion of subjects with apoB levels > 0.9 g/L as well as to calculate significance of interactions between smoking habits and the presence of hyperapoB genotypes on the relative odds (odds ratio (OR)) to exhibit plasma apoB concentrations > 0.9 mmol/L. Age and gender were included as covariates. All statistical analyses were performed with the SPSS package (20.0. Armonk, NY: IBM Corp).

## Results

**Fig. 1:**
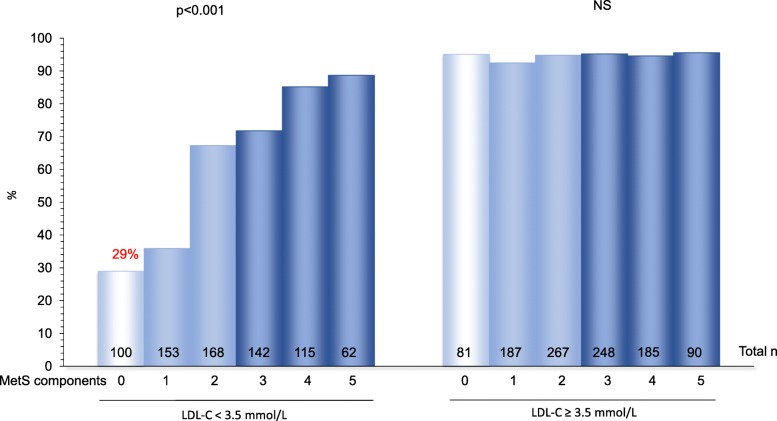
Proportion of subjects (%) with plasma apoB > 0.9 g/L, according to LDL-C and the number of metabolic syndrome components. Data were age-adjusted. NS = *P* > 0.1

As shown in Table 1, subjects with MetS were older, had higher concentrations of total apoB, an increased frequency of CAD (*P* < 0.001) and were more frequently carriers of a loss-of-function LPL gene mutation (*P* = 0.001). In both group, almost 50% of subjects were not carrying any of the mutation (or polymorphism) known to be associated with increase in apoB concentration. The proportion of subjects with plasma apoB > 0.9 g/L significantly rises with the number of MetS components but only when LDL-C is < 3.5 mmol/L (*P* < 0.001). Increase in the number of MetS components is associated with an OR of 1.8 (*P* < 0.001) to exhibit plasma apoB > 0.9 mmol/L among subjects with LDL-C < 3.5 mmol/L, whereas the relation between both variables is non-significant in subjects with LDL-C ≥ 3.5 mmol/L (OR = 1.1; *P* = 0.58). In the LDL-C < 3.5 mmol/L group, more than one fourth (29%) of individuals without any MetS component had plasma apoB > 0.9 g/L (Fig. 1).

**Table 1:** Subjects’ characteristics according to metabolic syndrome expression

	MetS (−) (*n* = 956)	MetS (+) (*n* = 842)	***P***-value
Age, years	47.2 ± 0.4	51.7 ± 0.3	< 0.001
Female, %	49.0	45.1	NS
Total cholesterol, mmol/L	6.3 ± 0.1	6.8 ± 0.1	< 0.001
Total triglyceride, mmol/L^a^	1.3 (1.0–1.9)	2.6 (1.9–3.9)	< 0.001
LDL-Cholesterol, mmol/L	4.1 ± 0.1	4.1 ± 0.1	NS
HDL-Cholesterol, mmol/L	1.32 ± 0.01	0.96 ± 0.01	< 0.001
Total apoB, g/L	1.12 ± 0.01	1.26 ± 0.01	< 0.001
CAD, %	24.6	41.1	< 0.001
Number of carried hyperapoB genotypes, %			NS
0	46.2	45.0	
1	41.8	40.1	
≥ 2	12.0	14.9	
PPARα-L162V, %	25.3	24.8	NS
Loss-of-function HeLPL, %	12.3	17.9	0.001
Apo e4 carrier or E2/2, %	29.3	28.7	NS
Apo e4 allele carrier	3.8	4.9	NS
Apo E2/2	25.5	23.8	NS
Smoking, %			NS
0–10 cigarette/day	80.8	80.9	
> 10 cigarette/day	19.2	19.1	

Mean ± SE unless otherwise specified; NS = *P* > 0.1; *CAD* Coronary artery disease, *MetS* Metabolic syndrome. HyperapoB genotypes = PPARα-L162V, heterozygous (He) loss-of-function LPL mutation, apo e4 allele or apo E2/2 genotype

^a^Median (Interquartile range)

As shown in Table 2, there is a significant interaction between the presence of ≥1 hyperapoB variants, in either the LPL, APOE, or PPARα genes, and smoking habits on the risk to exhibit apoB > 0.9 g/L, but only among subjects with a low risk profile, that is without MetS and with LDL-C < 3.5 mmol/L. Interestingly, this interaction remains significant even among individuals that never had CAD who are in primary prevention (Table 3).

**Table 2:** Interaction between smoking and the presence of ≥1 hyperapoB genotypes on the relative odds to exhibit apoB > 0.9 g/L, among subjects with LDL-C < 3.5 mmol/L, according to the expression of metabolic syndrome

	n	OR	95% CI	*P*-value
MetS (−)				
Smoking x HyperapoB genotypes	421	3.47	1.21–9.94	0.02
MetS (+)				
Smoking x HyperapoB genotypes	319	1.02	0.23–4.58	NS

Including age and gender as covariates; *MetS* Metabolic syndrome, *OR* Odds ratio, *NS P* > 0.1

**Table 3:** Interaction between smoking and the presence of ≥1 hyperapoB genotypes on the relative odds of apoB > 0.9 g/L, among subjects with LDL-C < 3.5 mmol/L without metabolic syndrome, according to CAD history

	n	OR	95% CI	*P*-value
CAD (−)				
Smoking x HyperapoB genotypes	345	3.73	1.09–12.78	0.04
CAD (+)				
Smoking x HyperapoB genotypes	76	2.92	0.28–31.01	NS

Including age and gender as covariates, *CAD*:Coronary artery disease, *OR* Odds ratio, *NS P* > 0.1

## Discussion

This study shows that a significant proportion of individuals have increased plasma apoB levels > 0.9 g/L even when LDL-C levels are not elevated and in absence of any criterion of the MetS. This observation is in accordance with previous results suggesting that not only can high apoB concentrations be observed in an important proportion of individuals with “optimal” LDL-C [34] but also that increases in cholesterol-depleted apoB particles can precede the onset of obesity. Hypertriglyceridemia and hyperglycemia (and then potentially the MetS) can therefore be an important marker of future CVD [35]. Current results are consistent with this statement. This study however goes a step further in the identification of potential markers of increased CVD risk, before the increase of apoB concentrations beyond high-risk levels. It also shows that common gene-smoking interactions are significantly associated with an increased risk of higher apoB concentrations among subjects with an apparently low-risk profile without history of CAD and who are in primary prevention.

The influence of smoking habits and of smoking x gene interactions on atherosclerotic processes, including inflammation, fat peroxidation, brown fat metabolism and coronary artery calcification has been relatively well documented in past years. Most studies get the same observation: smoking by itself is an important risk factor and, in addition, it significantly interacts with many gene variants by modulating the risk of CAD, CVD and stroke [19, 20]. Various studies have also suggested that smoking may influence apolipoproteins concentrations and lipoprotein particle sizes, although available results still remain limited and somewhat discordant [16, 17, 36]. Besides, there are fewer published results about gene x smoking interaction influence on lipid-lipoprotein metabolism, and there is even more disagreement between published observations [22, 23]. Interestingly, a recent large, multi-ancestry, genome-wide gene-smoking interaction study has reported 13 novel loci associated with lipid levels [37]. However, there are still apparent discrepancies between available results. Such a situation is not surprising considering the challenges associated with gene-environment interaction studies due to the difficulty to accurately control environment factors and to uniformly evaluate and characterize the studied disorders, especially for continuous traits such as lipid-lipoprotein phenotypes.

Study of subjects without apparent lipid-lipoprotein abnormalities or related disorders gave a particular clinical significance to the current results. They suggest that the assessment of life habits and the genotyping of some common gene variants may detect several patients with increased apoB measurements, and thus at a higher risk of CVD, that could have fallen of the radar with traditional guidelines. They also give more strength and solid arguments for preventive approaches, especially among people who do not believe themselves exposed to an increased CVD risk. This study included subjects whose average age was in the fifties and then who has been exposed to smoking for several years. Preventive strategies that could arise from such results could be more difficult to implement with subjects whose firmly fixed life habits may be more difficult to change. Besides, it has been shown that even a significant number of patients with proven coronary heart disease continue to smoke [38, 39]. However, results obtained from previous studies have shown that, even when young, smokers’ lipoproteins are altered as compared to non-smokers [40]. Although the present study was not conducted in young people, it could be hypothesize that similar results would be observed in younger subjects. It has been show that the cardiovascular risk associated with increased apoB levels would be higher among people under 50 [41]. Implementation of preventives strategies would therefore be of particular importance even among younger people.

The current results are in accordance with previous studies suggesting that smoking may alter the expression of genes influencing HDL metabolism or TG and LDL particle size [42]. Each of the genetic modulators included in this study, namely PPARα-L162V, heterozygous loss-of function LPL mutation, apo e4 allele or apo E2/2 genotype, is associated with a plasma apoB concentration increase, by acting on specific apoB-containing lipoproteins [7–9]. Various mechanisms could explain this interaction that could be partly due, for instance, to the unfavorable interaction between apo E4 and smoking, as well as to a reduced LPL activity previously observed among smokers [43–45]. Moreover, it has been shown that smoking is associated with an increased hepatic lipase activity [45, 46]. This increase, along with an elevation of TG-rich lipoprotein concentrations induced by the presence of the hyperapo B genotypes herein studied, could promote LDL-TG hydrolysis by hepatic lipase, which results in the generation of small-dense LDL particles and, then, in an increase of plasma apo B levels [47]. The epigenetic signature of smoking is also another interesting avenue to further investigate. In addition to the epigenetic effects of smoking on cancer physiopathology, there is increasing number of results showing significant associations between tobacco smoking and DNA methylation of genes associated with MetS components and associated risk [48].

The present study was conducted among a uniformly detailed phenotyped sample. This is one of its greatest strength. In addition, the French-Canadian population of the Saguenay–Lac-Saint-Jean region, from which the participants originate from, is another strength. This population is descended from a founder population that settled this region 300–400 years ago. This founder effect provides several benefits for studying the genomic determinants of complex traits. Genetic heterogeneity remains a problem in gene association studies that can be avoided by analyzing homogeneous populations that have a geographic stability and are most likely uniform in their environmental exposure [49].

One limitation of the current study is its modest sample size. It doesn’t allow the various subdivisions that would help to better document the associations observed, for instance a finer subdivision for the number of cigarettes smoked daily. Other life habits, including diet, which is clearly a central modulator in plasma lipid concentrations and can be related to smoking habits, was not available [50]. Finally, the cross-sectional design of the current study does not give any information about the potential causal pathway that may be implicated and the association with future CVD. Results should therefore be replicated in larger samples and diversified populations, with a further assessment of other life habits and, ideally, with the longitudinal follow-up of subjects to evaluate the hazard ratio of CVD.

## Conclusion

ApoB levels, now recognized as an efficient marker of CVD risk, could be beyond clinical cut-off levels among low-risk subjects without apparent lipid-lipoprotein abnormalities. Obtained results are in accordance with the fact that common genetic combinations involving key players in the VLDL catabolic pathway and their interactions with smoking habits, are associated with an increased risk of elevated apoB. In conclusion, the current study shows that the combination of the assessment of life habits and some common genes variants may detect a significant proportion of patients with increased apoB levels, and therefore at a higher risk of CVD, who could have been perceived as low-risk initially.

## Data Availability

The datasets used and/or analysed during the current study are available from the corresponding author on reasonable request.

## Abbreviations

Apo: Apolipoprotein
CAD: Coronary artery disease
CVD: Cardiovascular disease
FH: Familial hypercholesterolemia
HyperapoB: Hyperapobetalipoproteinemia
IDL: Intermediate-density lipoprotein
LDL-C: Low-density lipoprotein-cholesterol
LoFL: Loss-of-function
LPL: Lipoprotein lipase
MnCl2: Heparin and manganese chloride
Non-HDL-C: Non-high-density lipoprotein-cholesterol
OR: Odds ratio
PCR-RFLP: Polymerase chain reaction-restriction fragment length polymorphism
PPAR: Peroxisome proliferator-activated receptor
TG: Triglycerides
VLDL: Very-low-density lipoprotein
